# Trends in Proportions of Respiratory Syncytial Virus Infections Among Reported Respiratory Tract Infection Cases in Children Aged 0 to 5 Years in Western Pacific and Southeast Asia Regions: A Systematic Review and Meta‐Analysis

**DOI:** 10.1111/irv.70077

**Published:** 2025-02-08

**Authors:** Sangshin Park, Hyelan Lee, Jung Yoon Park, Sujin Choi, Hyun Jung Kim, Lorenzo Bertizzolo, Young Hwa Lee, Young June Choe

**Affiliations:** ^1^ Graduate School of Urban Public Health University of Seoul Seoul South Korea; ^2^ Department of Urban Big Data Convergence University of Seoul Seoul South Korea; ^3^ Medical Affairs Sanofi Pasteur Seoul South Korea; ^4^ Medical Evidence Generation Sanofi Lyon France; ^5^ Allergy Immunology Center Korea University Seoul South Korea; ^6^ Department of Pediatrics Korea University College of Medicine Seoul South Korea; ^7^ Korea University Anam Hospital Seoul South Korea

**Keywords:** Asia, burden, respiratory syncytial virus, RSV

## Abstract

**Background:**

Respiratory syncytial virus (RSV) is an important cause of bronchiolitis and pneumonia in children globally. This study aimed to incorporate new data to update estimates of RSV burden in children through 5 years of age in Western Pacific and Southeast Asia Regions.

**Methods:**

A systematic review and meta‐analysis were conducted to examine the proportion of RSV among cases of respiratory tract infection (RTI) in children in Western Pacific and Southeast Asia Regions using random effects models. Studies were eligible if they met the following inclusion criteria: (1) observational studies such as cohort and cross‐sectional studies; (2) studies on humans; (3) studies on patients with RTI or influenza‐like illness (ILI); (4) studies reporting incidence or proportion of RSV infection among respiratory related illness; and (5) studies on children aged 5 years or less.

**Findings:**

A total of 4403 studies were identified from an initial search. After screening titles, abstracts, and full‐text review, a total of 173 studies that met predefined eligibility criteria were included in the analysis. The overall proportion of RSV infections among all ARTIs was 18.7% (95% CI: 16.0%–21.5%), whereas the proportion of RSV infections among LRTIs was 28.7% (95% CI: 2.6%–30.3%) in children in Western Pacific and Southeast Asia Regions between 1970 and 2020. The proportion of RSV infections peaked in the 1980s at 33.4% (95% CI: 19.8%–48.5%), having increased from 10.6% (95% CI: 2.9%–22.2%) in the 1970s. It then showed a decreasing trend, with 28.9% (95% CI: 18.8%–40.3%) in the 1990s, 24.5% (95% CI: 22.3%–26.8%) in the 2000s, and 20.1% (95% CI: 17.8%–22.5%) in the 2010s. By country, Myanmar (50.0%; 95% CI, 47.5%–52.4%) and New Zealand (45.3%; 95% CI, 37.1%–53.7%) had the highest proportion during the overall time period, followed by Bhutan (45.2%; 95% CI, 36.4%–54.3%), Lao PDR (41.0%; 95% CI, 36.2%–46.0%), and Vietnam (35.5%; 95% CI, 19.3%–53.6%).

**Interpretation:**

Substantial RSV‐associated disease burden occurs in children in Western Pacific and Southeast Asia Regions. Our findings provide new and important evidence of the need for RSV prevention in Western Pacific and Southeast Asia countries. They could inform future preventive policy.

## 1 Introduction

Respiratory syncytial virus (RSV) is an important cause of bronchiolitis and pneumonia in infants globally. RSV is transmitted from person to person mainly by direct contact with respiratory droplets of infected people [1]. The clinical spectrum of RSV infection varies. Cases present with bronchiolitis in infancy show the highest proportion, followed by cases with viral pneumonia in toddlers [2]. RSV is also a major viral pathogen causing severe lung disease in adults, particularly the elderly [3].

East Asia and Pacific region houses approximately one third of the global population, including over a quarter of the world's children, totaling around 580 million children [4]. Notably, many countries in Asia report 2–10 times higher pneumonia cases (7–40 cases per 100 children annually) compared to the United States [5]. This higher burden of pneumonia in Asia is likely influenced by several factors, including resource limitations, strain on healthcare systems, and a lack of standardized pneumonia definitions [6]. These challenges can hinder the accurate estimation and comparison of pneumonia burden across different regions.

RSV is a significant contributor to lower respiratory tract infections (LRTIs) in children across the Western Pacific and Southeast Asian regions. However, comprehensive epidemiological data on RSV infections in children in these regions are limited, particularly regarding the trends and proportions of RSV among upper respiratory tract infections (URTIs) and LRTIs [7, 8, 9, 10, 11, 12]. Existing studies offer some insights into the impact of RSV. For example, in Japan, RSV was detected in 31% of children under 3 years hospitalized with acute respiratory tract infections (RTIs) [13]. In China, RSV has been identified in 40% of children with acute RTIs [14]. In Malaysia, RSV is the most commonly identified respiratory virus in children aged 6 months or younger, constituting 81.3% of LRTI cases [15]. However, a comprehensive understanding of RSV's epidemiology in Asian children remains incomplete. Research studies should be undertaken to elucidate the trend and proportion of RSV among URTIs and LRTIs, considering regional disparities of RSV seasonality in diverse Asian regions.

The lack of comprehensive data on health impact of RSV infection in the Asia Pacific region highlights the necessity for additional research to enhance comprehension of this disease's burden within this geographic area [16]. Thus, the present study aimed to conduct a systematic review and meta‐analysis to provide more comprehensive information regarding disease burden and epidemiology of RSV in children living in Western Pacific and Southeast Asian Regions. This review has potential to enable adjustments to clinical burden assessments by providing a more accurate estimation of the actual disease burden in Asian children.

## 2 Methods

### 2.1 Search Strategy

We adapted the methodology from the Meta‐Analysis of Observational Studies in Epidemiology [17] to perform this systematic review and meta‐analysis. We searched relevant articles using MEDLINE, EMBASE, CINAHL, and Global Health on December 2, 2022. We used keywords, thesaurus terms (e.g., MeSH and Emtree terms), and broad search criteria without language restriction (Table S1). We exploded each possible thesaurus term to obtain related subheadings. We limited the search to human studies and those published in English language. The following types of publications were excluded from the searching process: case reports, comment, letter, editorial, and review. Asian region of our interest included Western Pacific and Southeast Asia regions defined by the World Health Organization (WHO).

### 2.2 Study Selection and Data Extraction

Two reviewers (JP and HL) independently screened retrieved articles by screening titles and abstracts of articles, followed by full‐text evaluation after removing duplicated articles. Any disagreement in selection was resolved through arbitration by a third reviewer. Inclusion criteria were (1) observational studies such as cohort and cross‐sectional studies; (2) studies on humans; (3) studies on patients with RTI or influenza‐like illness (ILI); (4) studies reporting incidence or proportion of RSV infection among respiratory related illness; and (5) studies on children aged 5 years or less. When the study was specifically performed on LRTI patients, their data were classified separately from RTI patients and extracted. Therefore, data were extracted according to outcomes of RTI, LRTI, and ILI.

### 2.3 Risk of Bias and Quality Assessment

The quality of included studies was assessed by two independent reviewers based on the modified Newcastle‐Ottawa Scale (NOS) [18]. This modified NOS had the following three criteria: selection (clarity in describing the exposed population and inclusion criteria), comparability (clarity of case definition and exclusion criteria), and outcome (precision in explaining result reporting). We assigned a quality score of 1 if the study met each criterion or 0 if it did not. The resulting NOS rating ranged from 0 to 3, which provided an assessment of the quality of the study. Data quality was evaluated with a score of zero or one based on each of the following criteria: selection (clear description of exposed population and inclusion criteria for sampling), comparability (clarity of case definition and exclusion criteria), and outcome (precise explanation of result reporting). NOS rating ranged from zero to three.

### 2.4 Statistical Analysis

A meta‐analysis was performed to obtain pooled estimates of the proportion of RSV among RTI cases with 95% confidence intervals (CIs) in each decade (e.g., 1970s, 1980s, 1990s, 2000s, and 2010s). Pooled estimates were calculated after Freeman–Tukey Double Arcsine Transformation was performed to stabilize variances. Using score (Wilson) procedures, 95% CIs were calculated. A random‐effects model was used to pool the prevalence of RSV infection and Cochran's Q test with chi‐square statistics to test heterogeneity across the type of RSV infection or possible infection (e.g., RTI, LRTI, and ILI). All statistical analyses were performed using the “metaprop” package in Stata version 17.0 (College Station, TX, United States).

## 3 Results

**FIGURE 1:**
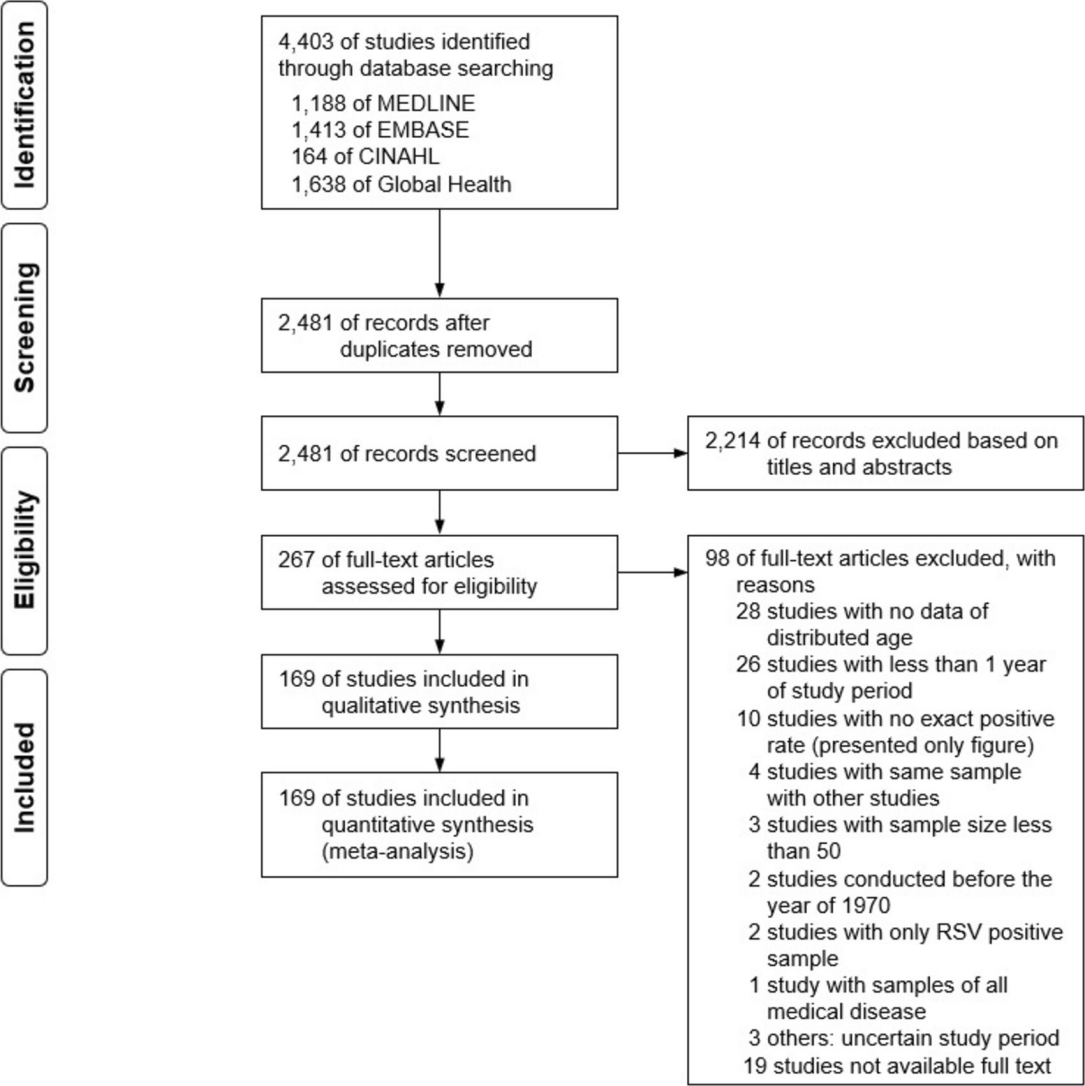
Selection of studies for systematic review and meta‐analysis.

**FIGURE 2:**
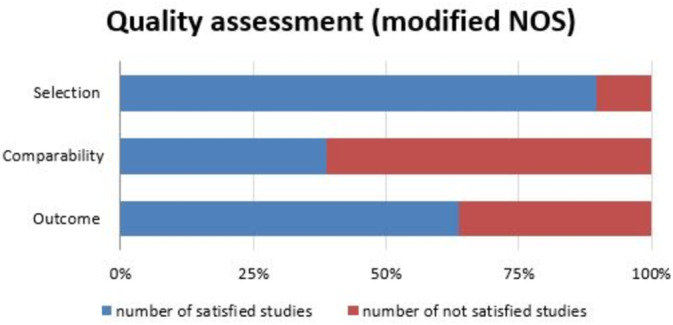
Results of modified risk‐of‐bias assessments.

A total of 4403 article citations were collected from the database. After removing duplicates, 2481 articles remained (Figure 1). Based on the titles and abstracts, 2214 studies were excluded from the review. Subsequently, a full‐text screening was conducted for 267 articles that met the eligibility criteria and were readily accessible. Of the 94 excluded articles, 28 lacked information on the participants' ages, 26 had a study duration of less than 1 year, and 10 presented the prevalence as images, making it difficult to determine the exact figures. Ultimately, 173 articles (469,658 patients) met our inclusion criteria for systematic review and meta‐analysis (Table 1). The agreement between articles selected by the two reviewers was moderate (Cohen's kappa coefficient = 0.75). After evaluating the quality of those 173 studies, 155 met the selection criteria, warranting a score of one. Under the second comparability criteria, 67 studies earned a score of one. For the final outcome criterion, 110 studies fulfilled the criteria, securing a score of one (Figure 2). Thirty‐two studies achieved full scores across all three criteria. Conversely, 14 studies received a score of 0 out of 3.

**TABLE 1:** Proportions of RSV infections in inpatient and outpatient settings.

Setting	No. of studies	No. of patients	No. of RSV positive	RSV positive, %
Inpatient	110	353,681	70,149	19.8
Outpatient/emergency department	29	66,130	7973	12.0
Both inpatient and outpatient/emergency department	23	318,68	7695	24.1
No information	11	179,79	3291	18.3
Total	173	469,658	89,036	19.0

**FIGURE 3:**
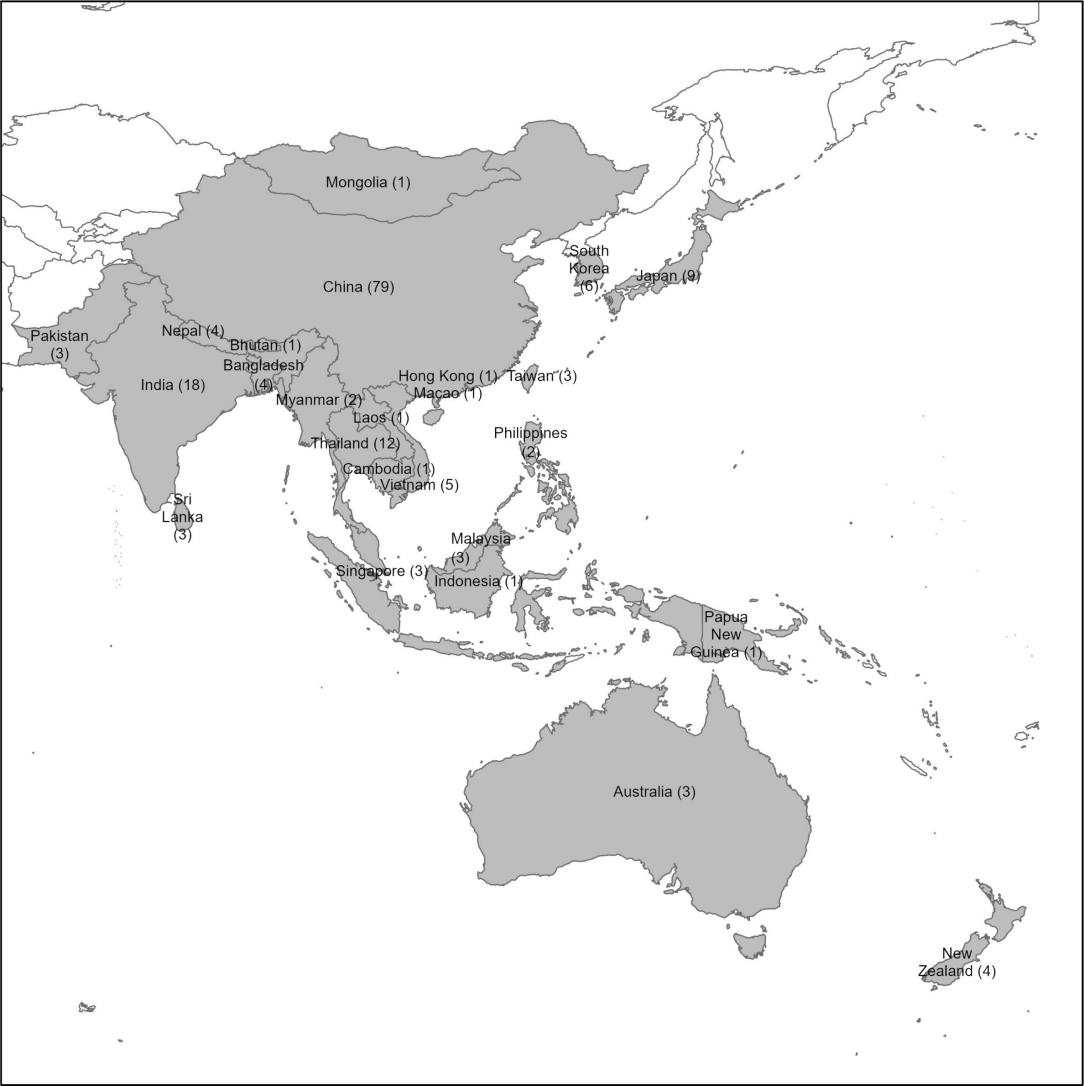
Distribution of included studies in Western Pacific and Southeast Asia Regions. *Note*: The number in parentheses next to the country name indicates the number of studies. Two studies were excluded from the map because they performed studies on several countries collectively.

Among 173 included studies, 50% (86/173) and 36% (63/173) were conducted in the 2010s and the 2000s, respectively, whereas the remaining 24 studies were conducted in the time period of the 1970s through 1990s. Most (*n* = 79) of studies were conducted in China, followed by those conducted in India (*n* = 18), Thailand (*n* = 12), Japan (*n* = 9), and South Korea (*n* = 6) (Figure 3). The remaining studies were performed in one or more of the 17 other countries in the Western Pacific or Southeast Asia regions. From the 1970s to the 2010s, 76 studies reported the proportion of RSV infection in RTI patients and 87 studies reported the proportion in LRTI patients. After the 2000s, 11 studies reported the proportion of RSV infection in ILI patients.

A prospective observational design was used the most as the study design (*n* = 80), followed by a retrospective observational design (*n* = 32) and a prospective cohort design (*n* = 30) (Table S2). A cross‐sectional design type was employed in 22 studies. A retrospective cohort design was used in six studies. Twenty‐nine (16.8%) studies with 66,130 (14.1%) patients were performed in outpatient or emergency department settings (Table 1). A total 469,658 patients were collectively included in our research. Proportions of RSV infection of patients in inpatient and outpatient/emergency department settings were 19.8% and 12.0%, respectively.

**FIGURE 4:**
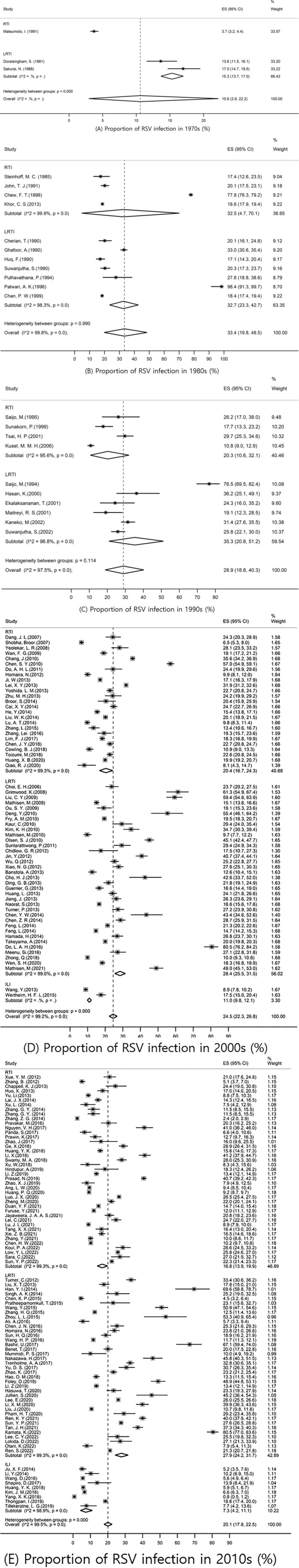
Proportion of RSV infection (A) in 1970s, (B) in 1980s, (C) in 1990s, (D) in 2000s, and (E) in 2010s. Abbreviations: ILI, influenza‐like illness; LRTI, lower respiratory tract infection; RSV, respiratory syncytial virus; RTI, respiratory tract infection.

**FIGURE 5:**
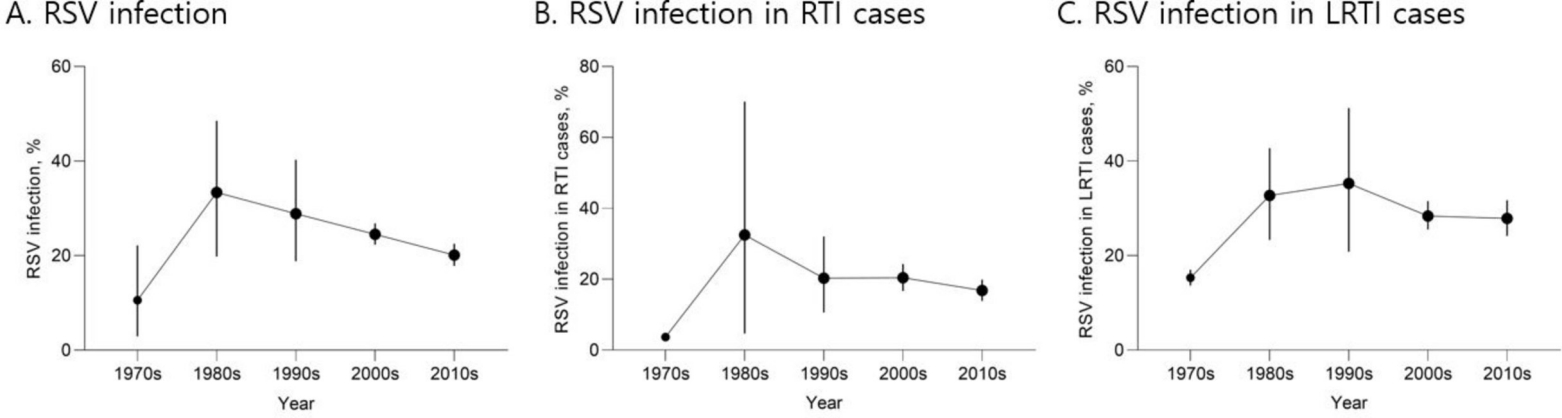
Summary of proportion of RSV infection from the 1970s to the 2010s. Abbreviations: LRTI, lower respiratory tract infection; RSV, respiratory syncytial virus; RTI, respiratory tract infection.

The pooled proportion of RSV among all RTIs was 18.7% (*n* = 185,786), whereas the proportion among LRTIs was 28.7% (*n* = 250,401). In the 1970s, the pooled proportion of RSV infection ranged from 3.7% (95% CI: 3.2%–4.4%) among RTIs to 17.0% (95% CI: 14.7%–19.5%) among LRTIs (Figure 4A). It was 10.6% (95% CI: 2.9%–22.2%) among all RTI patients (Figure 4A). In the 1980s, the pooled proportion of RSV infection was 32.5% (95% CI: 4.7%–70.1%) among RTIs and 32.7% (95% CI: 23.3%–42.7%) among LRTIs, with a pooled proportion of 33.4% (95% CI: 19.8%–48.5%) among all RTIs (Figure 4B). In the 1990s, the pooled proportion of RSV infection was 20.3% (95% CI: 10.6%–32.1%) among RTIs and 35.3% (95% CI: 20.8%–51.2%) among LRTIs, with a pooled proportion of 28.9% (95% CI: 18.8%–40.3%) among all RTIs (Figure 4C). In the 2000s, the pooled proportion of RSV infection was 20.4% (95% CI: 16.7%–24.3%) among RTIs, 28.4% (95% CI: 25.5%–31.5%) among LRTIs, and 11.0% (95% CI: 9.8%–12.1%) among ILI patients, with a pooled proportion of 24.5% (95% CI: 22.3%–26.8%) among all RTIs (Figure 4D). In the 2010s, the pooled proportion of RSV infection was 16.8% (95% CI: 13.9%–19.9%) among RTIs, 27.9% (95% CI: 24.2%–31.7%) among LRTIs, and 7.3% (95% CI: 4.2%–11.1%) among ILI patients, with a pooled proportion of 20.1% (95% CI: 17.8%–22.5%) among all RTIs (Figure 4E). The proportion of RSV infections peaked in the 1980s at 33.4% (95% CI: 19.8%–48.5%), having increased from 10.6% (95% CI: 2.9%–22.2%) in the 1970s. It then showed a decreasing trend, with 28.9% (95% CI: 18.8%–40.3%) in the 1990s, 24.5% (95% CI: 22.3%–26.8%) in the 2000s, and 20.1% (95% CI: 17.8%–22.5%) in the 2010s (Figure 5). The proportion of RSV infection in RTI patients decreased from 32.5% in the 1980s to 16.8% in the 2010s, whereas the proportion of RSV in LRTI patients decreased from 32.5% in the 1980s to 27.9% in the 2010s. By countries, excluding those studied only once, Myanmar (50.0%) and New Zealand (45.3%) had the highest proportions, followed by Vietnam (35.5%), Pakistan (32.2%), and Singapore (31.0%) (Figures S1 and S2).

## 4 Discussion

Our pooled analysis study findings indicate that RSV is a significant cause of reported acute lower respiratory disease, accounting for more than one in four cases reported in young children of Western Pacific and Southeast Asian countries. In addition, we observed a substantial number of RSV‐related hospitalizations in the underfive population. RSV was significantly more evident in LRTI cases than in RTI cases, although it might have been underestimated in countries with limited RSV disease surveillance resources [19]. Our study also found variations in the proportion of RSV infection over time, with an initial increase from the 1970s to 1990s and a subsequent decrease in the 2000s and 2010s [20, 21]. Several factors may have contributed to these temporal trends. The evolution of diagnostic methods, from antigen‐based assays to more sensitive PCR‐based techniques, could have influenced the detection and reporting of RSV infections. Additionally, the introduction of RSV prevention strategies, such as palivizumab prophylaxis for high‐risk infants, may have played a role in reducing the burden of RSV disease. Further surveillance is needed to clearly understand these trends and the changing epidemiology of RSV in Asian countries.

The 2019 Global Burden of Disease (GBD) study identified 3.6 million acute lower respiratory tract infection (ALRI) hospital admissions worldwide across children aged 0–5 years, including 1.4 million admissions of infants aged 0–6 months attributable to RSV [1]. That estimate derived from an attributable fraction assessment encompassing child at 23%–29% of all LRTIs, as established through a systematic review of published studies. It is notably similar to our estimated proportion of RSV among all RTI (25%), although our results were not specifically pertained to patients exclusively confined to hospitals. Notably, both approaches might have underestimated the proportional contribution of RSV among LRTI‐related hospitalizations due to the type of setting where RSV cases' finding and reporting might be limited. According to our assessment, RSV is linked to LRTIs with an annual range of 29%–34%. These figures are congruous with the burden of disease evaluations for RSV as outlined by the US Centers for Disease Control and Prevention [21].

The presented estimates aligned with prevailing global assessments of RSV‐related morbidity and mortality in infants [1]. Although prior research studies have typically presented the proportion or incidence of RSV in children through cross‐sectional snapshots, our study aimed to elucidate the evolving pattern of RSV incidences across multiple decades. This approach was undertaken to establish a comprehensive understanding of the desired objectives for RSV prevention in children across Western Pacific and Southeast Asian countries. Furthermore, our study expands existing epidemiological knowledge by furnishing a targeted appraisal specifically focused on children living in Western Pacific region [22]. Our current review and meta‐analysis aimed to enhance or progress our existing knowledge on RSV burden, which is constrained by the absence of published surveillance data, especially in South Asian region.

The burden of severe RSV‐associated diseases and subsequent hospitalizations in young children in Western Pacific and Southeast Asia regions is notably significant. Among these children, those with underlying chronic conditions have been identified as target risk groups for RSV prophylaxis, as outlined by the World Health Organization (WHO) [23]. Recent advent of various approaches for RSV prophylaxis in healthy infants, pregnant women, and elderly population provides options for preventing RSV in infants and young children [24, 25, 26]. Such initiatives hold promise of establishing and systemically evaluating routine immunization practices that can significantly heighten protection against severe RSV‐related illnesses. Nonetheless, it is important to acknowledge that these endeavors demand substantial investments in time and resources.

This analysis has several limitations. First, our estimates for LRTI episodes were established using a proportion of RSV‐positive cases derived from hospitalized illnesses. It is crucial to recognize that the broader category of LRTI episodes encompasses instances that may or may not require hospitalization. In settings with limited access to hospital services, individuals with nonhospitalized illnesses might share similar characteristics to those who do seek medical care. Consequently, the proportion associated with RSV could be comparable between hospitalized and nonhospitalized cases within these settings. However, this remains unknown in the absence of rigorous well‐designed studies conducted in out‐patient clinics and community settings. Second, our systematic review aggregated data spanning multiple years to assess the portion of RSV among RTIs and LRTIs within a given year. Pooling data in decade‐scale intervals may overestimate RSV incidence due to potential reinfections with different genotypes within that timeframe. However, the proportion of respiratory diseases attributed to RSV within a specific context or timeframe can be influenced by the lack of standardization in case finding and reporting, such as the type of specimen used for RSV testing, the clinical and laboratory criteria employed to define cases, the utilization of RSV prophylaxis, and the quality and completeness of the studies and surveillance conducted to provide attributable proportion data. Although suitable for our diverse set of studies, the NOS may not capture all potential sources of bias. Third, it is important to acknowledge that the proportion of children with respiratory illnesses hospitalized for those illnesses is subject to numerous influences, including severity of the respiratory illness, access to healthcare services, health‐seeking behaviors, diagnostic and referral practices, age, and underlying health conditions, all of which can exhibit variations among different populations and study sites. It is important to note that the observed decrease in RSV infection proportion in recent years may be partly attributed to diagnostic bias. The introduction and widespread use of multiplex PCR, which can identify a broader range of respiratory pathogens, could lead to a seemingly lower proportion of RSV infections simply due to the increased detection of other pathogens. This highlights the need for careful interpretation of our findings and emphasizes the importance of considering diagnostic methods when assessing RSV infection trends. Forth, the variability in laboratory tests used across studies (antigen detection, PCR, and viral culture) may have introduced heterogeneity and influenced the pooled RSV incidence estimates, despite employing a random‐effects model. Furthermore, previous analyses from the United States and France indicated that RSV positivity was often higher in cases with nonsevere LRI than in cases with severe LRI in children [27, 28]. Extrapolating the proportion of positive cases observed in hospitalized children to potentially milder cases of nonhospitalized LRI suggests that we might have underestimated the actual burden of RSV‐associated LRI episodes in Asian countries.

Our study highlights the need for robust surveillance data to guide effective RSV prevention and control strategies in Western Pacific and Southeast Asian regions. Standardized case definitions and unbiased surveillance are crucial for understanding the impact of RSV and other respiratory viruses.

## Author Contributions

**Sangshin Park:** data curation, supervision, formal analysis, methodology, writing – original draft. **Hyelan Lee:** methodology, software, formal analysis, validation, investigation. **Jung Yoon Park:** methodology, software, formal analysis, validation, investigation. **Sujin Choi:** methodology, validation, investigation. **Hyun Jung Kim:** methodology, conceptualization, resources, supervision, writing – review and editing, validation. **Lorenzo Bertizzolo:** conceptualization, methodology, validation, supervision, resources, writing – review and editing. **Young Hwa Lee:** formal analysis, project administration, supervision, data curation, resources, visualization, writing – original draft. **Young June Choe:** writing – original draft, funding acquisition, investigation, conceptualization, methodology, supervision, resources, formal analysis, project administration, writing – review and editing, validation.

## Conflicts of Interest

Sangshin Park, Hyelan Lee, Jung Yoon Park, and Young Hwa Lee have nothing to disclose. Young June Choe received research grant from Sanofi. Sujin Choi, Hyunjung Kim, and Lorenzo Bertizzolo are Sanofi employees and may hold shares and/or stock options in the company.

### Peer Review

The peer review history for this article is available at https://www.webofscience.com/api/gateway/wos/peer‐review/10.1111/irv.70077.

## Supporting information


**Table S1** Searching terms
**Table S2.** Description of included studies

**Figure S1** Proportion of RSV in China

**Figure S2** Proportion of RSV in Western Pacific and Southeast Asia Regions excluding China

## Data Availability

The data that support the findings of this study are available from the corresponding author upon reasonable request.
