# Supplementary material for: Trends in Proportions of Respiratory Syncytial Virus Infections Among Reported Respiratory Tract Infection Cases in Children Aged 0 to 5 Years in Western Pacific and Southeast Asia Regions: A Systematic Review and Meta‐Analysis
Source: Influenza Other Respir Viruses. 2025 Feb 8;19(2):e70077. doi: 10.1111/irv.70077 (PMC11806376; doi:10.1111/irv.70077)
Supplement: Supplementary file 1 — Table S1 Searching terms Table S2. Description of included studies [file IRV-19-e70077-s003.doc]

**Supplementary Table 1.** Searching terms

| # | Searches |
| --- | --- |
| MEDLINE through Ovid | |
| 1 | exp Respiratory Syncytial Viruses/ or exp Respiratory Syncytial Virus Infections/ or 'Respiratory Syncytial Virus*'.mp. |
| 2 | exp Asia/ or exp Asians/ or exp Oceania/ or ('Asia*' or 'Western Pacific' or 'Samoa*' or 'Australia*' or 'Bangladesh*' or 'Bhutan*' or 'Brunei*' or 'Cambodia*' or ('China' or 'Chinese') or 'Cook Island*' or 'Korea*' or 'Fiji*' or 'Polynesia*' or 'Guam*' or 'Hong Kong' or 'India*' or 'Indonesia*' or 'Japan*' or 'Kiribat*' or 'Lao*' or 'Maca*' or 'Maldiv*' or 'Malay*' or 'Marshall Island*' or 'Micronesi*' or 'Mongolia*' or ('Myanmar' or 'Burm*') or 'Nauru*' or 'Nepal*' or 'New Caledonia*' or 'New Zealand*' or 'Niue*' or 'Northern Mariana Island*' or 'Palau*' or 'Papua New Guinea*' or 'Philippin*' or 'Pitcairn Island*' or 'Samoa*' or 'Singapor*' or 'Solomon Island*' or 'Sri lanka*' or 'Thai*' or 'Timor*' or 'Tokelau*' or 'Tong*' or 'Tuvalu*' or 'Vanuatu*' or ('Viet nam*' or 'Vietnam*') or "Wallis and Futuna*" or 'Taiwan*').mp. |
| 3 | exp Child/ or exp Infant/ or ('child*' or 'infant*' or 'toddler*' or 'newborn*' or 'neonate*' or 'pediatric*').mp. |
| 4 | (Case Reports or Comment or Letter or Editorial or Review or Congress).pt. |
| 5 | (1 and 2 and 3) not 4 |
| 6 | limit 5 to humans |
| 2. EMBASE through Elsevier | |
| 1 | 'human'/de |
| 2 | ‘Human respiratory syncytial virus’/exp OR ‘respiratory syncytial virus infection’/exp OR ‘Respiratory Syncytial Virus*':ti,ab,lnk,kw |
| 3 | 'asia'/exp OR 'asian'/exp OR 'oceania'/exp OR 'oceanian'/exp OR ('Asia*' or 'Western Pacific' or 'Samoa*' or 'Australia*' or 'Bangladesh*' or 'Bhutan*' or 'Brunei*' or 'Cambodia*' or 'China' or 'Chinese' or 'Cook Island*' or 'Korea*' or 'Fiji*' or 'Polynesia*' or 'Guam*' or 'Hong Kong' or 'India*' or 'Indonesia*' or 'Japan*' or 'Kiribat*' or 'Lao*' or 'Maca*' or 'Maldiv*' or 'Malay*' or 'Marshall Island*' or 'Micronesi*' or 'Mongolia*' or 'Myanmar' or 'Burm*' or 'Nauru*' or 'Nepal*' or 'New Caledonia*' or 'New Zealand*' or 'Niue*' or 'Northern Mariana Island*' or 'Palau*' or 'Papua New Guinea*' or 'Philippin*' or 'Pitcairn Island*' or 'Samoa*' or 'Singapor*' or 'Solomon Island*' or 'Sri lanka*' or 'Thai*' or 'Timor*' or 'Tokelau*' or 'Tong*' or 'Tuvalu*' or 'Vanuatu*' or 'Viet nam*' or 'Vietnam*' or "Wallis and Futuna*" or 'Taiwan*'):ti,ab,lnk,kw |
| 4 | 'child'/exp OR ('child*' or 'infant*' or 'toddler*' or 'newborn*' or 'neonate*' or 'pediatric*'):ti,ab,lnk,kw |
| 5 | ('case reports' OR comment OR letter OR editorial OR review OR 'conference abstract'):it |
| 6 | (1 and 2 and 3 and 4) not 5 |
| 3. CINHAL through EBSCOhost | |
| S1 | MH "Respiratory Syncytial Viruses" OR MH "Respiratory Syncytial Virus Infections" OR TI ‘Respiratory Syncytial Virus*’ OR AB ‘Respiratory Syncytial Virus*’ OR MH ‘Respiratory Syncytial Virus*’ OR ‘Respiratory Syncytial Virus*’ [Expanders - Apply equivalent subjects] |
| S2 | MH "Pacific Islands+" OR MH "Australia+" OR MH "Asians+" OR TI ('Asia*' or ‘Oceania*’ or 'Western Pacific' or 'Samoa*' or 'Australia*' or 'Bangladesh*' or 'Bhutan*' or 'Brunei*' or 'Cambodia*' or 'China' or 'Chinese' or 'Cook Island*' or 'Korea*' or 'Fiji*' or 'Polynesia*' or 'Guam*' or 'Hong Kong' or 'India*' or 'Indonesia*' or 'Japan*' or 'Kiribat*' or 'Lao*' or 'Maca*' or 'Maldiv*' or 'Malay*' or 'Marshall Island*' or 'Micronesi*' or 'Mongolia*' or 'Myanmar' or 'Burm*' or 'Nauru*' or 'Nepal*' or 'New Caledonia*' or 'New Zealand*' or 'Niue*' or 'Northern Mariana Island*' or 'Palau*' or 'Papua New Guinea*' or 'Philippin*' or 'Pitcairn Island*' or 'Samoa*' or 'Singapor*' or 'Solomon Island*' or 'Sri lanka*' or 'Thai*' or 'Timor*' or 'Tokelau*' or 'Tong*' or 'Tuvalu*' or 'Vanuatu*' or 'Viet nam*' or 'Vietnam*' or "Wallis and Futuna*" or 'Taiwan*') OR AB ('Asia*' or ‘Oceania*’ or 'Western Pacific' or 'Samoa*' or 'Australia*' or 'Bangladesh*' or 'Bhutan*' or 'Brunei*' or 'Cambodia*' or 'China' or 'Chinese' or 'Cook Island*' or 'Korea*' or 'Fiji*' or 'Polynesia*' or 'Guam*' or 'Hong Kong' or 'India*' or 'Indonesia*' or 'Japan*' or 'Kiribat*' or 'Lao*' or 'Maca*' or 'Maldiv*' or 'Malay*' or 'Marshall Island*' or 'Micronesi*' or 'Mongolia*' or 'Myanmar' or 'Burm*' or 'Nauru*' or 'Nepal*' or 'New Caledonia*' or 'New Zealand*' or 'Niue*' or 'Northern Mariana Island*' or 'Palau*' or 'Papua New Guinea*' or 'Philippin*' or 'Pitcairn Island*' or 'Samoa*' or 'Singapor*' or 'Solomon Island*' or 'Sri lanka*' or 'Thai*' or 'Timor*' or 'Tokelau*' or 'Tong*' or 'Tuvalu*' or 'Vanuatu*' or 'Viet nam*' or 'Vietnam*' or "Wallis and Futuna*" or 'Taiwan*') OR MH ('Asia*' or ‘Oceania*’ or 'Western Pacific' or 'Samoa*' or 'Australia*' or 'Bangladesh*' or 'Bhutan*' or 'Brunei*' or 'Cambodia*' or 'China' or 'Chinese' or 'Cook Island*' or 'Korea*' or 'Fiji*' or 'Polynesia*' or 'Guam*' or 'Hong Kong' or 'India*' or 'Indonesia*' or 'Japan*' or 'Kiribat*' or 'Lao*' or 'Maca*' or 'Maldiv*' or 'Malay*' or 'Marshall Island*' or 'Micronesi*' or 'Mongolia*' or 'Myanmar' or 'Burm*' or 'Nauru*' or 'Nepal*' or 'New Caledonia*' or 'New Zealand*' or 'Niue*' or 'Northern Mariana Island*' or 'Palau*' or 'Papua New Guinea*' or 'Philippin*' or 'Pitcairn Island*' or 'Samoa*' or 'Singapor*' or 'Solomon Island*' or 'Sri lanka*' or 'Thai*' or 'Timor*' or 'Tokelau*' or 'Tong*' or 'Tuvalu*' or 'Vanuatu*' or 'Viet nam*' or 'Vietnam*' or "Wallis and Futuna*" or 'Taiwan*') OR ('Asia*' or ‘Oceania*’ or 'Western Pacific' or 'Samoa*' or 'Australia*' or 'Bangladesh*' or 'Bhutan*' or 'Brunei*' or 'Cambodia*' or 'China' or 'Chinese' or 'Cook Island*' or 'Korea*' or 'Fiji*' or 'Polynesia*' or 'Guam*' or 'Hong Kong' or 'India*' or 'Indonesia*' or 'Japan*' or 'Kiribat*' or 'Lao*' or 'Maca*' or 'Maldiv*' or 'Malay*' or 'Marshall Island*' or 'Micronesi*' or 'Mongolia*' or 'Myanmar' or 'Burm*' or 'Nauru*' or 'Nepal*' or 'New Caledonia*' or 'New Zealand*' or 'Niue*' or 'Northern Mariana Island*' or 'Palau*' or 'Papua New Guinea*' or 'Philippin*' or 'Pitcairn Island*' or 'Samoa*' or 'Singapor*' or 'Solomon Island*' or 'Sri lanka*' or 'Thai*' or 'Timor*' or 'Tokelau*' or 'Tong*' or 'Tuvalu*' or 'Vanuatu*' or 'Viet nam*' or 'Vietnam*' or "Wallis and Futuna*" or 'Taiwan*') [Expanders - Apply equivalent subjects] |
| S3 | MH "Child+" OR TI ('child*' or 'infant*' or 'toddler*' or 'newborn*' or 'neonate*' or 'pediatric*' ) OR AB ( 'child*' or 'infant*' or 'toddler*' or 'newborn*' or 'neonate*' or 'pediatric*') OR MH ('child*' or 'infant*' or 'toddler*' or 'newborn*' or 'neonate*' or 'pediatric*') OR ('child*' or 'infant*' or 'toddler*' or 'newborn*' or 'neonate*' or 'pediatric*') [Expanders - Apply equivalent subjects] |
| S4 | S1 and S2 and S3 |
| S5 | S1 and S2 and S3 [Limiters - Publication Type: Abstract, Case Study, Commentary, Editorial, Letter, Review] |
| S6 | S4 not S5 [Limiters – Human] |
| 4. Global Health through EBSCOhost | |
| S1 | DE "Human respiratory syncytial virus" OR TI Respiratory Syncytial Virus* OR AB Respiratory Syncytial Virus* OR Respiratory Syncytial Virus* OR SU Respiratory Syncytial Virus* [Expanders - Apply equivalent subjects] |
| S2 | (DE "Asia" OR DE "Central Asia" OR DE "East Asia" OR DE "South Asia" OR DE "South East Asia" OR DE "West Asia" OR DE "Asians" OR DE "Chinese" OR DE "Oceania" OR DE "Australasia" OR DE "Micronesia" OR DE "Polynesia") OR TI ( 'Asia*' or ‘Oceania*’ or 'Western Pacific' or 'Samoa*' or 'Australia*' or 'Bangladesh*' or 'Bhutan*' or 'Brunei*' or 'Cambodia*' or 'China' or 'Chinese' or 'Cook Island*' or 'Korea*' or 'Fiji*' or 'Polynesia*' or 'Guam*' or 'Hong Kong' or 'India*' or 'Indonesia*' or 'Japan*' or 'Kiribat*' or 'Lao*' or 'Maca*' or 'Maldiv*' or 'Malay*' or 'Marshall Island*' or 'Micronesi*' or 'Mongolia*' or 'Myanmar' or 'Burm*' or 'Nauru*' or 'Nepal*' or 'New Caledonia*' or 'New Zealand*' or 'Niue*' or 'Northern Mariana Island*' or 'Palau*' or 'Papua New Guinea*' or 'Philippin*' or 'Pitcairn Island*' or 'Samoa*' or 'Singapor*' or 'Solomon Island*' or 'Sri lanka*' or 'Thai*' or 'Timor*' or 'Tokelau*' or 'Tong*' or 'Tuvalu*' or 'Vanuatu*' or 'Viet nam*' or 'Vietnam*' or "Wallis and Futuna*" or 'Taiwan*') OR AB ( 'Asia*' or ‘Oceania*’ or 'Western Pacific' or 'Samoa*' or 'Australia*' or 'Bangladesh*' or 'Bhutan*' or 'Brunei*' or 'Cambodia*' or 'China' or 'Chinese' or 'Cook Island*' or 'Korea*' or 'Fiji*' or 'Polynesia*' or 'Guam*' or 'Hong Kong' or 'India*' or 'Indonesia*' or 'Japan*' or 'Kiribat*' or 'Lao*' or 'Maca*' or 'Maldiv*' or 'Malay*' or 'Marshall Island*' or 'Micronesi*' or 'Mongolia*' or 'Myanmar' or 'Burm*' or 'Nauru*' or 'Nepal*' or 'New Caledonia*' or 'New Zealand*' or 'Niue*' or 'Northern Mariana Island*' or 'Palau*' or 'Papua New Guinea*' or 'Philippin*' or 'Pitcairn Island*' or 'Samoa*' or 'Singapor*' or 'Solomon Island*' or 'Sri lanka*' or 'Thai*' or 'Timor*' or 'Tokelau*' or 'Tong*' or 'Tuvalu*' or 'Vanuatu*' or 'Viet nam*' or 'Vietnam*' or "Wallis and Futuna*" or 'Taiwan*') OR ( 'Asia*' or ‘Oceania*’ or 'Western Pacific' or 'Samoa*' or 'Australia*' or 'Bangladesh*' or 'Bhutan*' or 'Brunei*' or 'Cambodia*' or 'China' or 'Chinese' or 'Cook Island*' or 'Korea*' or 'Fiji*' or 'Polynesia*' or 'Guam*' or 'Hong Kong' or 'India*' or 'Indonesia*' or 'Japan*' or 'Kiribat*' or 'Lao*' or 'Maca*' or 'Maldiv*' or 'Malay*' or 'Marshall Island*' or 'Micronesi*' or 'Mongolia*' or 'Myanmar' or 'Burm*' or 'Nauru*' or 'Nepal*' or 'New Caledonia*' or 'New Zealand*' or 'Niue*' or 'Northern Mariana Island*' or 'Palau*' or 'Papua New Guinea*' or 'Philippin*' or 'Pitcairn Island*' or 'Samoa*' or 'Singapor*' or 'Solomon Island*' or 'Sri lanka*' or 'Thai*' or 'Timor*' or 'Tokelau*' or 'Tong*' or 'Tuvalu*' or 'Vanuatu*' or 'Viet nam*' or 'Vietnam*' or "Wallis and Futuna*" or 'Taiwan*') [Expanders - Apply equivalent subjects] |
| S3 | (DE "children" OR DE "boys" OR DE "children with disabilities" OR DE "foster children" OR DE "girls" OR DE "latchkey children" OR DE "adopted children" OR DE "preschool children" OR DE "school children" OR DE "infants" OR DE "neonates") OR TI ('child*' or 'infant*' or 'toddler*' or 'newborn*' or 'neonate*' or 'pediatric*') OR AB('child*' or 'infant*' or 'toddler*' or 'newborn*' or 'neonate*' or 'pediatric*') OR ('child*' or 'infant*' or 'toddler*' or 'newborn*' or 'neonate*' or 'pediatric*') [Expanders - Apply equivalent subjects] |
| S4 | S1 and S2 and S3 |
| S5 | S1 and S2 and S3 [Limiters - Publication Type: Correspondence, Editorial] |
| S6 | S4 not S5 |

| # | Searches |
| --- | --- |
| 1 | 'human'/de |
| 2 | ‘Human respiratory syncytial virus’/exp OR ‘respiratory syncytial virus infection’/exp OR ‘Respiratory Syncytial Virus*':ti,ab,lnk,kw |
| 3 | 'asia'/exp OR 'asian'/exp OR 'oceania'/exp OR 'oceanian'/exp OR ('Asia*' or 'Western Pacific' or 'Samoa*' or 'Australia*' or 'Bangladesh*' or 'Bhutan*' or 'Brunei*' or 'Cambodia*' or 'China' or 'Chinese' or 'Cook Island*' or 'Korea*' or 'Fiji*' or 'Polynesia*' or 'Guam*' or 'Hong Kong' or 'India*' or 'Indonesia*' or 'Japan*' or 'Kiribat*' or 'Lao*' or 'Maca*' or 'Maldiv*' or 'Malay*' or 'Marshall Island*' or 'Micronesi*' or 'Mongolia*' or 'Myanmar' or 'Burm*' or 'Nauru*' or 'Nepal*' or 'New Caledonia*' or 'New Zealand*' or 'Niue*' or 'Northern Mariana Island*' or 'Palau*' or 'Papua New Guinea*' or 'Philippin*' or 'Pitcairn Island*' or 'Samoa*' or 'Singapor*' or 'Solomon Island*' or 'Sri lanka*' or 'Thai*' or 'Timor*' or 'Tokelau*' or 'Tong*' or 'Tuvalu*' or 'Vanuatu*' or 'Viet nam*' or 'Vietnam*' or "Wallis and Futuna*" or 'Taiwan*'):ti,ab,lnk,kw |
| 4 | 'child'/exp OR ('child*' or 'infant*' or 'toddler*' or 'newborn*' or 'neonate*' or 'pediatric*'):ti,ab,lnk,kw |
| 5 | ('case reports' OR comment OR letter OR editorial OR review OR 'conference abstract'):it |
| 6 | (1 and 2 and 3 and 4) not 5 |

**Supplementary Table 2.** Description of included studies

| Study | Study period | Study design | Age | Setting | Test | Outcome | Sample (n) | RSV (n) |
| --- | --- | --- | --- | --- | --- | --- | --- | --- |
| China (n=79) |  |  |  |  |  |  |  |  |
| Liu, C. Y. et al. (2009) | 2007.03-2008.02 | Prospective cohort | 1 month-5 years | INP | RT-PCR | ALRTI | 446 | 265 |
| Ou, S. Y. et al. (2009) | 2007.01-2007.12 | Prospective cohort | 0-5 years | INP | PCR | ALRTI | 345 | 66 |
| Wan, F. G. et al. (2009) | 2007.07-2008.06 | Prospective observational study | 1 month-7 years | INP | RT-PCR | ARTI | 1455 | 304 |
| Chang, J. et al. (2010) | 2007.01-2008.12 | Prospective observational study | 13 days-13 years | INP | DIF | ARI | 4793 | 1704 |
| Chen, S. Y. (2010) | 2005.01-2008.12 | Retrospective analysis | 0-13 years | OPD | DIF | RTI | 3300 | 1255 |
| Deng, Y. et al. (2010) | 2006-2008 (winter) | Prospective observational study | 0-3 years | INP | RT-PCR, direct fluorescent assay | Bronchiolitis | 112 | 62 |
| Zou, L. R. et al. (2011) | 2006.09-2009.09 | Prospective observational study | All ages | NA | Fluorescence Quota PCR | ARI | 503 | 109 |
| Jin, Y. et al. (2012) | 2006.12-2009.12 | Prospective observational study | 0-14 years | INP | PCR or RT-PCR | ALRTI | 746 | 318 |
| Wu, Q. et al. (2012) | 2005.10-2007.10 | Case-control study | Children* | INP | DIF & IIF assay | ALRI | 1194 | 301 |
| Xiao, N. G. et al. (2012) | 2007.09-2008.08 | Prospective observational study | 0-14 years | INP | RT-PCR | ALRTI | 1123 | 310 |
| Xue, Y. M. (2012) | 2010.04-2011.03 | Prospective observational study | 1 month-14 years | INP | ELISA | RTI | 496 | 104 |
| Zhang, B. et al. (2012) | 2011.01-2011.12 | Prospective observational study | 0-14 years | OPD & INP | Immunofluorescence | RTI | 668 | 34 |
| Ding, G. B. et al. (2013) | 2008-2012 | Prospective observational study | 0-6 years | INP | Indirect ELISA | ALRTI | 774 | 169 |
| Huang, L. et al. (2013) | 2006.06-2011.03 | Prospective observational study | 5 days-14 years | INP | DIF assay | LRTI | 1244 | 300 |
| Huo, X. et al. (2013) | 2011.01-2011.12 | Surveillance for SARI | < 5 years | INP | RT-PCR | SARI | 511 | 87 |
| Ji, W. et al. (2013) | 2009.09-2011.10 | Prospective observational study | All ages | INP | DIF assay, RT-PCR | ARL | 9198 | 1573 |
| Jiang, J. et al. (2013) | 2006.06-2011.03 | Prospective observational study | Children* | INP | DIF assay | ALRTI | 963 | 253 |
| Lei, X. Y. et al. (2013) | 2009-2011 | Retrospective analysis | < 17 years | INP | DIF assay | RTI | 17500 | 5581 |
| Liu, X. T. et al. (2013) | 2011.04-2012.033 | Prospective observational study | 1 month-9 years | INP | Fluorescent immunoassay | CAP | 625 | 111 |
| Wang, Y. et al. (2013) | 2008.07-2012.06 | Retrospective observational study | < 2 years | INP | PCR | ILI | 2122 | 189 |
| Yu, L. et al. (2013) | 2012.07-2013.06 | Prospective observational study | 0-14 years | OPD | IIF assay | ARTI | 1549 | 136 |
| Zhu, M. H. et al. (2013) | 2006.09-2009.08 | Prospective observational study | 45- days-14 years | OPD & INP | Quantitative fluorescence PCR | RTI | 554 | 118 |
| Cai, X. Y. et al. (2014) | 2007.10-2011.08 | Prospective observational study | 30 days-12 years | INP | Multiplex PCR | ARTI | 1868 | 431 |
| Chen, Z. R. et al. (2014) | 2009.01-2010.12 | Prospective observational study | 1-24 months | INP | DIF assay | Bronchiolitis | 998 | 286 |
| Feng, L. et al. (2014) | 2009.01-2013.09 | Surveillance for ALRI | All ages | INP | RT-PCR | ALRI | 17127 | 2520 |
| Feng, L. et al. (2014) | 2009-2012 | Prospective observational study | 0-4 years | INP | PCR or RT-PCR | Pneumonia | 4508 | 962 |
| He, Y. et al. (2014) | 2007.07-2010.06 | Prospective observational study | 45 days-14 years | INP | Monoplex PCR, RVP assay | ARTI | 1815 | 280 |
| Ju, X. F. et al. (2014) | 2011.07-2013.07 | Prospective observational study | All ages | INP | RT-PCR | ILI | 461 | 24 |
| Lai, J. X. et al. (2014) | 2013.05-2014.02 | Cross-sectional study | 0-6 years | INP | DIF assay | RTI | 1144 | 164 |
| Li, Y. et al. (2014) | 2011 | Cross-sectional study | All ages | OPD | RT-PCR | ILI | 215 | 22 |
| Liu, W. K. et al. (2014) | 2009.07-2012.06 | Cross-sectional study | 0-14 years | No information | RT-PCR | ARI | 3713 | 748 |
| Xu, L. et al. (2014) | 2010.07-2012.07 | Cross-sectional study | Children* | INP | RT-PCR | RI | 91 | 8 |
| Zhang, G. Y. et al. (2014) | 2013.01-2013.12 | Cross-sectional study | 0-14 years | NA | IF assay | ARTI | 321 | 37 |
| Zhang, Z. G. et al. (2014) | 2012.03-2013.03 | Cross-sectional study | 0-14 years | OPD | IIF assay | ARTI | 1209 | 130 |
| Chen, K. P. et al. (2015) | 2011.08-2013.08 | Cross-sectional study | 0-14 years | INP | Pneumoslide IgM technology | CAP | 661 | 30 |
| Wang, Y. et al. (2015) | 2010.01-2012.12 | Retrospective observational study | 0-2 years | INP | PCR | Bronchiolitis | 674 | 343 |
| Zhang, H. Q. et al. (2015) | 2013.01-2013.12 | Cross-sectional study | 0-11 years | INP | DIF & antiviral specific monoclonal  antibody | LRTI | 3407 | 425 |
| Zhang, L. et al. (2015) | 2009-2013 | Cross-sectional study | All ages | OPD & INP | Multiple PCR | RTI | 471 | 89 |
| Zhou, L. L. et al. (2015) | 2012-2014 (winter) | Prospective observational study | 0-2 years | INP | Real-Time qPCR | Bronchiolitis | 60 | 32 |
| Chen, J. N. et al. (2016) | 2014.01-2014.12 | Prospective observational study | 1 month-15 years | INP | PCR | CAP | 487 | 123 |
| Sun, H. Q. et al. (2016) | 2012.12-2014.11 | Prospective cohort | < 5 years | INP | DIF assay | Wheezing | 709 | 134 |
| Wang, D. et al. (2016) | 2011.04-2014.03 | Prospective observational study | < 5 years | OPD | RT- PCR, PCR | ILI | 3662 | 206 |
| Wang, H. P. et al. (2016) | 2012.01-2015.12 | Prospective cohort | < 14 years | INP | DIF assay | ARI | 28875 | 3008 |
| Zhang, L. et al. (2016) | 2009.01-2014.12 | Prospective cohort | All ages | OPD & INP | PCR | RI | 383 | 74 |
| Dang, J. L. et al. (2017) | 2009-2013 | Retrospective cohort | < 3 years | INP | Multiplex PCR | ARI | 374 | 91 |
| Yu, D. S. et al. (2017) | 2012.07-2013.07 | Prospective observational study | < 5 years | INP | PCR | ALRTI | 391 | 120 |
| Zhao, J. et al. (2017) | 2015.08-2016.07 | Prospective observational study | All ages | INP | RT-PCR | SARI | 81 | 13 |
| Zhao, K. et al. (2017) | 2011.01-2016.12 | Retrospective observational | < 6 years | INP | DIF assay | ALRTI | 1602 | 372 |
| Chen, J. Y. et al. (2018) | 2009.09-2014.02 | Cross-sectional study | 1 month-15 years | INP | Primers and multiplex PCR | ARI | 1764 | 401 |
| Ge, X. et al. (2018) | 2010.01-2016.12 | Prospective observational study | < 4 years | INP | Multiplex real-time PCR | ARI | 1205 | 348 |
| Hao, O. M. et al. (2018) | 2015.01-2015.12 | Prospective observational study | 6 month-14 years | INP | Immunofluorescence | CAP | 1169 | 156 |
| Huang, Y. K. et al. (2018) | 2011.04-2014.03 | Prospective surveillance | < 5 years | OPD & INP | RT-PCR, DIF assay | ILI/ SARI | 3267  1838 | 192  287 |
| Li, X. et al. (2018) | 2011.01-2015.11 | Prospective active surveillance | All ages | OPD & INP | PCR or RT-PCR | ARI | 803 | 98 |
| Swamy, M. A. et al. (2018) | 2012-2016 | Prospective observational study | 0-5 years | INP | multiplex RT-PCR | ARTI | 997 | 279 |
| Xu, W. et al. (2018) | 2015.01-2016.12 | Cross-sectional study | All ages | INP | RT-PCR, Serodia Myco II gelatin  particle agglutination test | SARI | 96 | 8 |
| Yang, X. K. et al. (2018) | 2016.01-2016.12 | Prospective observational study | 0-5 years | INP | Fluorescent quantitative PCR | ILI | 77 | 16 |
| Zhong, Q. (2018) | 2009.01-2015.12 | Retrospective cohort | 0-28 days | INP | DIF assay | Pneumonia | 8128 | 810 |
| Li, Z. et al. (2019) | 2017.01-2017.12 | Prospective observational study | 1-14 years | INP | DIF assay | RI | 2240 | 301 |
| Zhao, X. J. et al. (2019) | 2014.08-2017.12 | Prospective observational study | All ages | OPD & INP | PCR | RI | 318 | 19 |
| Huang, P. Q. et al. (2020) | 2019.01-2019.12 | Retrospective | 0-14 years | INP | DIF | ARTI | 14267 | 1310 |
| Huang, X. B. et al. (2020) | 2009.01-2018-12 | Retrospective observational | All ages | No information | In-house real-time PCR | ARI | 10366 | 2068 |
| Li, X. M. et al. (2020) | 2017.04-2018.03 | Retrospective observational study | 1 month-5years | INP | DIF assay | CAP | 685 | 273 |
| Li, Y. L. et al. (2020) | 2017.12-2018.11 | Prospective observational study | All ages | OPD | RT-PCR, fluorescent assay | AFRS | 404 | 37 |
| Liu, J. et al. (2020) | 2014.06-2019.05 | Retrospective observational case-control | 0-14 years | INP | IIF assay | Pneumonia | 5425 | 481 |
| Luo, J. X. et al. (2020) | 2017.03-2019.02 | Prospective observational study | 1-14 years | OPD | Double amplification method (DAT) | RTI | 6646 | 764 |
| Qiao, R. J. et al. (2020) | 2016-2019 | Cross-sectional observational | 3 month-12 years | No information | Real-time fluorescent  quantitative PCR | ARI | 111 | 9 |
| Wen, S. H. et al. (2020) | 2008.01-2017.12 | retrospective review | < 18 years | INP | DIF assay | LRTI | 78033 | 15634 |
| Zheng, M. et al. (2020) | 2018-02-2019.01 | Prospective observational study | 0-6 years | INP | DIF assay | RTI | 1674 | 369 |
| Duan, Y. F. et al. (2021) | 2018.03-2020.03 | Retrospective observational study | 0-10 years | OPD | DIF assay | ARTI | 9708 | 1423 |
| Lu, J. L. et al. (2021) | 2019.01-2020.08 | Retrospective observational study | Children | OPD | DIF assay | RI | 3842 | 288 |
| Ren, K. Y. et al. (2021) | 2013.06-2018.05 | Retrospective observational study | < 2 years | INP | Multiplex PCR assays | ALRI | 2066 | 826 |
| Sun, Y. P. et al. (2021) | 2014.10-2017.09 | Retrospective review | < 2 years | INP | DIF assay | Pneumonia | 5581 | 1541 |
| Tan, J. H. et al. (2021) | 2011.11-2018.11 | Retrospective observational study | < 2 years | INP | DIF assay &PCR | ARTI | 1012 | 377 |
| Tang, X. X. et al. (2021) | 2018-2019 | Cross-sectional study | < 15 years | OPD | RT-PCR | RTI | 379 | 62 |
| Xie, Z. B. et al. (2021) | 2017.10-2020.10 | Prospective observational study | < 5 years | INP | Fluorescence quantitative PCR | SARI | 1335 | 220 |
| Zhang, Y. et al. (2021) | 2016.01-2018.12 | Cross-sectional study | < 2 years | INP | DIF assay | RTI | 1483 | 149 |
| Chen, H. W. et al. (2022) | 2019.01-2019.12 | Retrospective analysis | < 16 years | INP | DIF assay | RTI | 12955 | 1324 |
| Ren, S. et al. (2022) | 2010.01-2014.14 | Retrospective observational study | < 5 years | INP | DIF assay | ALRI | 19317 | 4107 |
| Sun, Y. P. et al. (2022) | 2014.10-2017.09 | Retrospective observational study | < 2 years | INP | DIF assay | ARTI | 7248 | 1618 |
| India (n=18) |  |  |  |  |  |  |  |  |
| Steinhoff, M. C. et al. (1985) | 1981.12-1982.11 | Retrospective observational study | 1 month-5 years | OPD | Neutralizing sera | ARI | 184 | 2 |
| Cherian, T. et al. (1990) | 1985.02-1987.12 | Prospective observational study | < 5 years | OPD & INP | IIF assay | LRI | 323 | 65 |
| John, T. J. et al. (1991) | 1982.02-1987.12 | Prospective observational study | < 6 years | INP | IIF assay | ARI | 809 | 163 |
| Patwari, A. K. et al. (1996) | 1988.09-1989.08 | Prospective observational study | < 12 years | INP | IF assay & EIA | Bronchopneumonia/ Pneumonia | 61 | 30 |
| Maitreyi, R. S. (2000) | 1995.09-1997.04 | Prospective cohort | 7 days-60 months | OPD, ER | IIF assay | ALRTI | 89 | 17 |
| Shobha, B. (2007) | 2001.10-2005.03 | Prospective cohort | < 3 years | OPD | IIF assay | ARI | 281 | 83 |
| Kaur, C. et al. (2010) | 2007.01-2007.12 | Prospective observational study | 1-12 months | No information | Viral culture, ELISA, PCR | Bronchiolitis | 245 | 72 |
| Yeolekar, L. R. et al. (2010) | 2002-2004 | Prospective observational study | All ages | INP | IF assay | ARTI | 331 | 93 |
| Broor, S. et al. (2014) | 2009.08-2011.07 | population-based surveillance | < 5 years | INP | RT-PCR | RTI | 245 | 50 |
| Singh, A. K. et al. (2014) | 2011.06-2012.05 | Prospective observational study | 0-14 years | INP | Mono/multiples RT-PCR | ALRI | 155 | 39 |
| Meenu, S. et al. (2016) | 2008.04-2011.03 | Prospective observational study | 3-59 months | OPD & INP | ELISA | CAP | 377 | 102 |
| Pravakar, M. et al. (2016) | 2013.08-2013.09 | Prospective observational study | 2-60 months | OPD | Mono/multiplex RT-PCR | ARI | 300 | 61 |
| Mummidi, P. S. et al. (2017) | 2013.08-2014.08 | Prospective observational study | < 5 years | INP | Multiplex real-time PCR assay | LRTI with wheezing | 70 | 7 |
| Panda, S. et al.(2017) | 2012.11-2014.10 | Prospective observational study | < 13 years | OPD | Multiplex PC | ARTI | 228 | 15 |
| Prawin, K. et al. (2017) | 2012.08-2014.12 | prospective cohort study | < 12 months | INP | Multiplex RT-PCR | ARI | 395 | 50 |
| Hindupur, A. et al. (2019) | 2016.04-2017.04 | Prospective pilot study | All ages | No information | Real time RT-PCR | ARI | 120 | 22 |
| Koul, P. A. et al. (2022) | 2013.10-2014.09 | Prospective observational study | < 5 years | INP | Duplex RT-PCR | SARI | 412 | 118 |
| Sara, C. et al. (2022) | 2019.09-2020.02 | Prospective observational study | < 5 years | INP | Multiplex RT-PCR | ARTI | 256 | 69 |
| Thailand (12) |  |  |  |  |  |  |  |  |
| Sunakorn, P. et al. (1990) | 1998-1999 | Prospective observational study | < 5 years | INP | Fluorescent antibody techniques | SARI | 226 | 40 |
| Suwanjutha, S. et al. (1990) | 1986.01-1987.12 | Prospective observational study | < 5 years | INP | IF assay | ALRI | 596 | 121 |
| Puthavathana, P. et al. (1994) | 1987.01-1987.12  1989.06-1990.05 | Prospective observational study | < 6 months | OPD & INP | IIF assay | Pneumonia | 76 | 31 |
| Ekalaksananan, T. et al. (2001) | 1992.08-1994.11 | Prospective observational study | < 5 years | INP | IF assay, EIA | ALRI | 74 | 18 |
| Suwanjutha, S. et al. (2002) | 1998.11-2001.02 | Prospective cohort | < 5 years | OPD | IF staining | LRTI | 472 | 122 |
| Fry, A. M. et al. (2010) | 2003-09-2007.12 | Retrospective cohort | All ages | INP | RT PCR paired-sera from a subset of patients with IgG enzyme immunoassay | ALRT | 4103 | 799 |
| Olsen, S. J. et al. (2010) | 2003.09-2005.12 | [Surveillance]  Retrospective cohort | All ages | INP | Culture, RT–PCR, ELISA (IgG) | Pneumonia | 1325 | 498 |
| Suntarattiwong, P. et al. (2011) | 2007.12-2009.08 | Prospective observational study | 0-1 year | INP | RT-PCR | ALRTI | 354 | 104 |
| Naorat, S. et al. (2013) | 2008.01-2011.12 | Retrospective cohort  [population-based surveillance] | all ages | INP | rRT PCR | ALRI | 4839 | 802 |
| Turner, P. et al. (2013) | 2009.04-2011.09 | Prospective observational study | All ages | INP | rRT PCR | Pneumonia | 640 | 174 |
| Pratheepamornkull, T. et al. (2015) | 2013.06-2015.05 | Prospective observational study  [Surveillance] | 1-5 months | No information | RT-PCR, in-house PCR assay | CAP | 91 | 21 |
| Thongpan, I. et al. (2019) | 2016.01-2017.12 | Retrospective analysis | All ages | OPD & INP | Multiplex one-step RT-PCR | ILI | 3458 | 644 |
| Japan (9) |  |  |  |  |  |  |  |  |
| Sakurai, N. et al. (1988) | 1979.04-1986.10 | Prospective observational study | 0-14 years | OPD | CF test | LRTI | 920 | 156 |
| Matsumoto, I. et al. (1991) | 1973.09-1983.12 | Prospective cohort | 0-14 years | OPD | Sensitivity tests on BUDR,  chloroform, and acid for CPE  positive strains | RTI | 3746 | 139 |
| Saijo, M. et al. (1994) | 1991.04-1993.03 | Prospective observational study | 0-3 months | INP | Enzyme immunoassay | Acute bronchiolitis | 162 | 124 |
| Saijo, M. et al. (1995) | 1993.04-1994.03 | Prospective observational study | 0-3 months | INP | Test Pack RSV™ | RTI | 65 | 17 |
| Kaneko, M. et al. (2002) | 1997.07-2000.06 | Retrospective observational | < 3 years | INP | ELFA or EIA | LRTI | 535 | 168 |
| Hamada, H. et al. (2014) | 2007.04-2012.03 | Prospective observational study | < 2 years | INP | RT-PCR | LRTD | 721 | 193 |
| Takeyama, A. et al. (2014) | 2008.02-2009.08 | Prospective observational study | 0-3 years | INP | PCR | LRTI, wheezing | 412 | 114 |
| Nakazawa, H. et al. (2017) | 2013.10-2014.02 | Prospective observational study | 0-5 years | INP | PCR | ALRTI | 301 | 138 |
| Hasuwa, T. et al. (2020) | 2013.02-2015.01 | Prospective observational study | < 5 years | INP | Multiplex PCR assays | ALRI | 373 | 87 |
| South Korea (6) |  |  |  |  |  |  |  |  |
| Choi, E. H. et al. (2006) | 2000.09-2005.08 | Prospective observational study | 0-5 years | OPD & INP | Multiplex RT-PCR, IF assay | LRTI | 515 | 122 |
| Kim, K. H. et al. (2010) | 2008.09-2009.03 | Prospective observational study | < 5 years | INP | Multiplex RT-PCR | LRTI | 418 | 145 |
| Cho, H. J. et al. (2013) | 2009.01-2010.05 | Prospective cohort | < 1 month | INP | Multiplex RT-PCR, | ALRI | 108 | 46 |
| Han, Y. I. et al. (2014) | 2011.10-2013.04 | Prospective cohort | < 6 months | INP | Multiplex RT- PCR assays | ALRTI | 79 | 55 |
| Kim, J. M. et al. (2018) | 2013-2015 | Prospective observational study  [Surveillance] | All ages | OPD | rRT PCR | URTI | 16842 | 1116 |
| Lee, E. et al. (2020) | 2010.01-2015.12 | Retrospective chart review | < 18 years | INP | RT-PCR | RTI | 23242 | 6051 |
| Vietnam (5) |  |  |  |  |  |  |  |  |
| Do, A. H. L. et al. (2011) | 2004.11-2008.01 | Prospective descriptive study | 2months-13 years | INP | Mono/multiplex RT-PCR | ARI | 295 | 72 |
| Yoshida, L. M. et al. (2013) | 2007.04-2010.03 | Cross-sectional survey  Followed by case-control study | 0-5 years | INP | Multiplex PCR | ARI | 1768 | 401 |
| Do, L. A. H. et al. (2016) | 2009.05-2010.12 | Prospective observational | 1-24 months | INP | Multiplex RT-PCR | LRTI | 375 | 302 |
| Toizumi, M. et al. (2018) | 2007.01-2012.04 | Cross-sectional study | < 2 years | INP | Multiplex RT-PCR | ARI | 1941 | 439 |
| Pham, H. T. et al. (2020) | 2015.01-2017.03 | Cross-sectional study | 1 month-5 years | INP | Immune chromate graphic method  RT-PCR | Pneumonia | 202 | 59 |
| Bangladesh (4) |  |  |  |  |  |  |  |  |
| Huq, F. et al. (1990) | 1986.06-1988.05 | Prospective observational study | < 5 years | INP | IF assay | ALRTI | 601 | 103 |
| Hasan, K. et al. (2006) | 1993.10-1994.09 | Prospective cohort | 0-2 years | INP | ELISA | ALRI | 58 | 21 |
| Homaira, N. et al. (2012) | 2009.04-2011.03 | Prospective cohort | < 2 years | OPD | rRT-PCR | ARI | 918 | 91 |
| Homaira, N. et al. (2016) | 2010-01-2014.12 | Prospective cohort  [prospective hospital-based  surveillance & community-based health care utilization survey] | < 5 years | INP | rRT PCR | ALRI | 829 | 197 |
| Nepal (4) |  |  |  |  |  |  |  |  |
| Mathisen, M. et al. (2009) | 2004.07-2007.06 | Cross-sectional study | 2-35 months | OPD | Multiplex RT- PCR assay | Pneumonia | 2219 | 334 |
| Mathisen, M. et al. (2010) | 2006.03-2007.06 | Case-control study | 2-35 months | OPD | Multiplex RT- PCR assay | Pneumonia | 680 | 66 |
| Banstola, A. et al. (2013) | 2008.07-2011.08 | Descriptive cross-sectional study | < 5 years | INP | NA | Pneumonia | 772 | 97 |
| Mathisen, M. et al. (2021) | 2006.01-2008.06 | Retrospective analysis | 2-35 months | OPD | Updated PCR assay | Pneumonia | 610 | 299 |
| NZ (4) |  |  |  |  |  |  |  |  |
| Grimwood, K. et al. (2008) | 2003-2005 | Retrospective observational study | < 24 months | INP | DIF assay, RT-PCR, nested PCR | Bronchiolitis | 230 | 141 |
| Trenholme, A. A. et al. (2017) | 2019.08-2011.07 | Prospective observational study | < 2 years | INP | Automated multiplex PCR | LRI | 1645 | 540 |
| Foley, D. et al. (2019) | 2014.01-2015.12 | Retrospective case review | < 2 years | INP | IMAGEN Respiratory Syncytial Virus qualitative immunofluorescence  test, Quidel Sofia RSV  immunofluorescence | Bronchiolitis | 556 | 272 |
| Prasad, N. et al. (2019) | 2012.04-2015.12 | Retrospective review | < 5 years | INP | RT-PCR | LRTI | 3923 | 1597 |
| Pakistan (3) |  |  |  |  |  |  |  |  |
| Ghafoor, A. et al. (1990) | 1986.11-1988.03 | Prospective observational study | < 5 years | OPD, INP | Viral culture, IF assay | ALRTI | 1492 | 491 |
| Ali, A. et al. (2016) | 2011.10-201.06 | Prospective community-based  active surveillance | 0-2 years | No information | Multiplex PCR assays | ARI | 230 | 13 |
| Bashir, U. et al. (2017) | 2011.03-2012.04 | Prospective observational study | < 2 years | INP | RT-PCR | ALRI | 155 | 102 |
| AUS (3) |  |  |  |  |  |  |  |  |
| Kusel, M. M. H. et al. (2006) | 1996.07-1999.07 | Prospective cohort | 0-1 year | OPD | RT-PCR | ARI | 976 | 106 |
| Chappell, K. J. et al. (2013) | 2012.01-2012.12 | Cross-sectional study | < 5 years | INP | Quantitative PCR & culture | ARI | 201 | 49 |
| Lim, F. J. et al. (2017) | 2008-2012 | Prospective cohort | 6-59 months | INP | Viral culture, multiplex tandem PCR | ARI (febrile) | 2356 | 431 |
| Malaysia (3) |  |  |  |  |  |  |  |  |
| Chan, P. W. et al. (1999) | 1982.01-1997-12 | Retrospective review | 2 weeks  -24 months | INP | Tissue culture, IF assay | LRTI | 5691 | 1047 |
| Khor, C. S. et al, ( 2013) | 1982-2008 | Retrospective observational study | 0-5 years | INP | IF assay, viral Isolation | RTI | 10269 | 1913 |
| Low, Y. L. et al. (2022) | 2015.01-2019.12 | Retrospective observational study | 0-18 years | OPD & INP | Multiplex PCR | ARI | 5391 | 1390 |
| Singapore (3) |  |  |  |  |  |  |  |  |
| Doraisingham, S. et al. (1981) | 1977.01-1979.12 | Retrospective cohort | < 12 years | OPD & INP | IIF assay | LRTS, ILI | 880 | 116 |
| Chew, F. T. et al. (1998) | 1990.09-1994.09 | Retrospective observational study | All ages | OPD & INP | Viral isolation, IF assay,  complement fixation test | RTI | 3369 | 2565 |
| Ang, L. W. et al. (2020) | 2014-2018 | Prospective observational study | All ages | OPD | rRT PCR | ARI | 3855 | 363 |
| Sri Lanka (3) |  |  |  |  |  |  |  |  |
| Shapiro, D. et al. (2017) | 2013.03-2015.01 | Cross-sectional study | >= 1 year | OPD | Multiplex PCR | LRI | 101 | 14 |
| Tillekeratne, L. G. et al. (2019) | 2012.06-2014.10 | Prospective observational study | All ages | INP | rRT PCR | AFI | 130 | 10 |
| Jayaweera, J. A. A. S. et al. (2021) | 2013.03-2014.08 | Prospective cross-sectional study | 1 month-5 years | INP | IF assay | ARI | 861 | 179 |
| Taiwan (3) |  |  |  |  |  |  |  |  |
| Tsai, H. P. et al. (2001) | 1997.01-1999.12 | Prospective observational study | < 12 years | OPD & INP | IF assay | ARTI | 367 | 109 |
| Chen, Y. W. et al. (2014) | 2009.01-2011.03 | Prospective observational study | < 2 years | INP | Immunofluorescent assay | Bronchiolitis | 113 | 49 |
| Lee, C. Y. et al. (2022) | 2014.10-2017.06 | Prospective observational study | < 2years | INP | xTAG RVP Fast Assay v2.0 | Bronchiolitis | 184 | 47 |
| Myanmar (2) |  |  |  |  |  |  |  |  |
| Turner, C. et al. (2012) | 2017 | Prospective cohort | < 2 years | OPD | RT-PCR | Pneumonia | 1085 | 362 |
| Kamata, K. et al. (2022) | 2017.05-2019.04 | Prospective observational study | 1 month-12 years | INP | Rapid test, RT-PCR | ALRI | 559 | 450 |
| Philippines (2) |  |  |  |  |  |  |  |  |
| Furuse, Y. et al. (2021) | 2014.03-2016.06 | Prospective cohort | < 5 years | OPD & INP | PCR, RT-PCR | ARI | 4735 | 566 |
| Otani, K. et al. (2022) | 2014.03-2016.06 | Prospective cohort | < 5 years | OPD & INP | Multiplex Real-time PCR | LRTI | 329 | 26 |
| Bhutan (1) |  |  |  |  |  |  |  |  |
| Jullien, S. et al. (2020) | 2017.07-2018.06 | Prospective cohort | 2-59 months | INP | Molecular screening  (multiplex RT-PCT QIA Stat respiratory panel) | Pneumonia | 115 | 52 |
| Cambodia (1) |  |  |  |  |  |  |  |  |
| Guerrier, G. et al. (2013) | 2007.04-2010.02 | Prospective observational study | < 1 year | INP | Multiplex RT-PCR/  polymerase chain reaction | LRTI | 1006 | 167 |
| Hong Kong (1) |  |  |  |  |  |  |  |  |
| Cowling, B. J. et al. (2018) | 2007-20=10 | Prospective observational study | 0-16 years | OPD | xTAG RVP FAST multiplex assay | ARI | 822 | 90 |
| Indonesia (1) |  |  |  |  |  |  |  |  |
| Lokida, D. et al. (2022) | 2017.07-2019.09 | Prospective observational study | 2-59 months | INP | PCR | CAP | 188 | 51 |
| Lao PDR (1) |  |  |  |  |  |  |  |  |
| Nguyen, V. H. et al. (2017) | 2013.12-2014.12 | Prospective cohort | < 5years | INP | Multiplexed Taqman real-time PCRs | ALRTI | 383 | 15 |
| Macao (1) |  |  |  |  |  |  |  |  |
| Lei, C. et al. (2021) | 2014.01-2017.12 | Retrospective observational study | < 13 years | INP | xMAP multiplex assays | ARI | 4486 | 94 |
| Mongolia (1) |  |  |  |  |  |  |  |  |
| Lu, A. T. et al. (2014) | 2009.01-2012.10 | Retrospective observational study | < 14 years | No information | rRT-PCR | ARI | 2199 | 178 |
| PNG (1) |  |  |  |  |  |  |  |  |
| Chidlow, G. R. et al. (2012) | 2005.05-2009.03 | Prospective observational study | < 18 months | No information | Multiplex RT-PCR | ALRI | 80 | 14 |
| Muti-countries (2) |  |  |  |  |  |  |  |  |
| Wertheim, H. F. L. et al. (2015) | 2008.07-2009.06 | Retrospective review | All ages | INP | Polymerase chain reaction. | ILI | 776 | 136 |
| Benet, T. et al. (2017) | 2010.05-2014.06 | Prospective case-control study | < 5 years | OPD | RT-PCR | Pneumonia | 888 | 178 |

AFI: acute febrile infection, AFRS: acute febrile respiratory symptoms, ALR/ALRI: acute lower respiratory infection, ARI: acute respiratory infection, ALRTI: acute lower respiratory tract infection, ARTI: acute respiratory tract infection, CAP: community acquired pneumonia, CF: complement fixation, DIF: direct immunofluorescent, DTA: double amplification, EIA: enzyme immune assay, ELFA: enzyme-linked fluorescent immunoassay, EIA: enzyme immunoassay, ELISA: enzyme-linked immunosorbent assay, ER: emergency room, IF: indirect immunofluorescent, ILI: influenza-like illness, INP: inpatient, LRI: lower respiratory infection, LRTD: lower respiratory tract disease, LRTI: lower respiratory tract infection, OPD: outpatient department, PCR: polymerase chain reaction, rRT-PCR: real-time reverse-transcription polymerase chain reaction, RI: respiratory infection, RVP: respiratory viral panel, RTI: respiratory tract infection, RT-PCR: reverse Transcription Polymerase Chain Reaction, SARI: severe acute respiratory infection, URTI: upper respiratory tract infection.

* Children: Age range is not presented in the context.

* Outcome categorization: RTI (AFRS, ARI, ARTI, RI, RTI, SARI), LRTI (ALR/ALRI, ALRTI, LRI, LRTD, LRTI, CAP, Bronchiolitis), Others (AFI, ILI, URTI)

* Only data from children aged 5 years and under were utilized for our analysis.

**Supplementary Figure Legends**

**Supplementary Figure 1.** Proportion of RSV in China

**Supplementary Figure 2.** Proportion of RSV in Western Pacific and Southeast Asia Regions excluding China
