# Supplementary figures and images for: Trends in Proportions of Respiratory Syncytial Virus Infections Among Reported Respiratory Tract Infection Cases in Children Aged 0 to 5 Years in Western Pacific and Southeast Asia Regions: A Systematic Review and Meta‐Analysis
Source: Influenza Other Respir Viruses. 2025 Feb 8;19(2):e70077. doi: 10.1111/irv.70077 (PMC11806376; doi:10.1111/irv.70077)

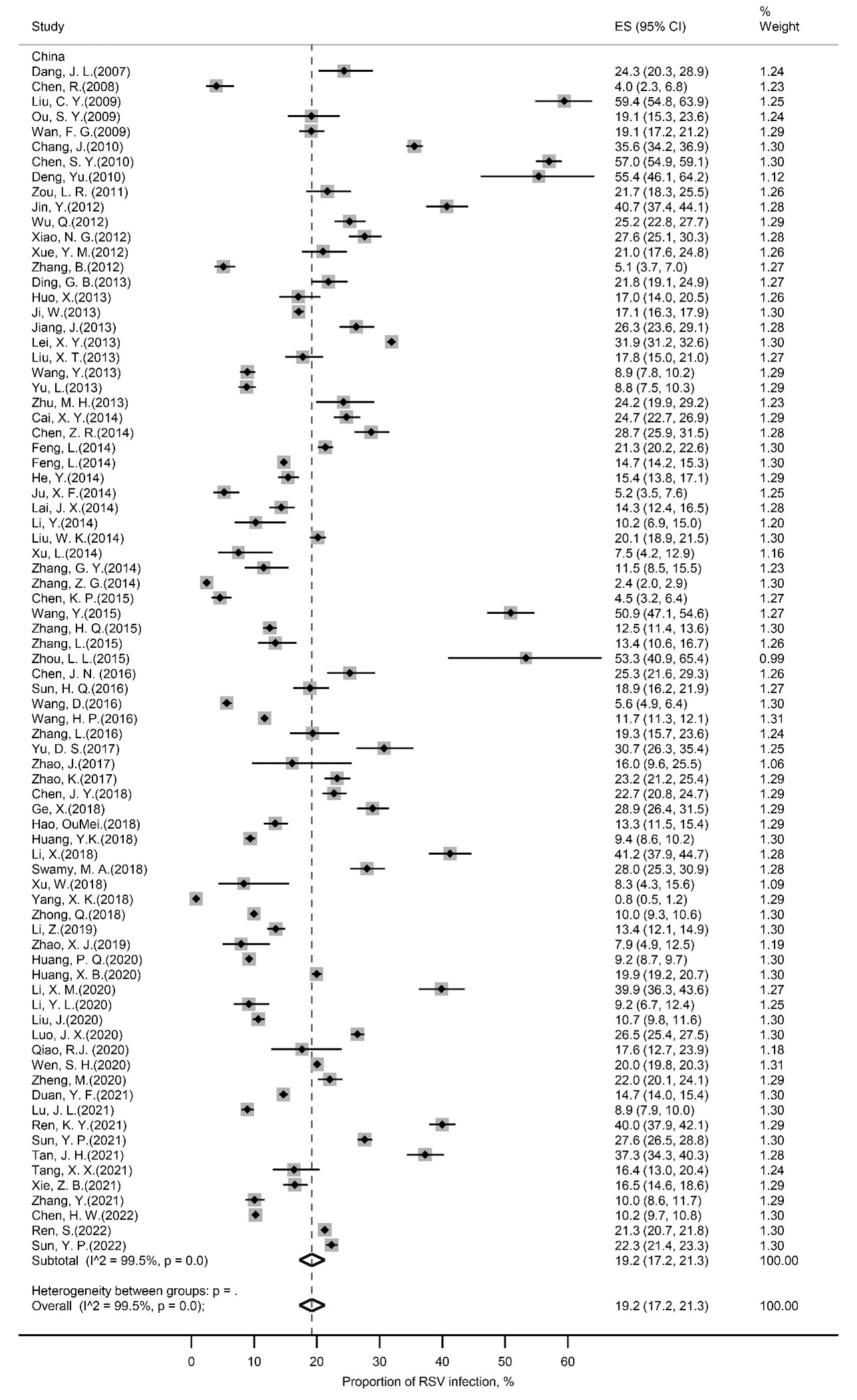

Supplement: Supplementary file 2 — Figure S1 Proportion of RSV in China [file IRV-19-e70077-s001.jpg]

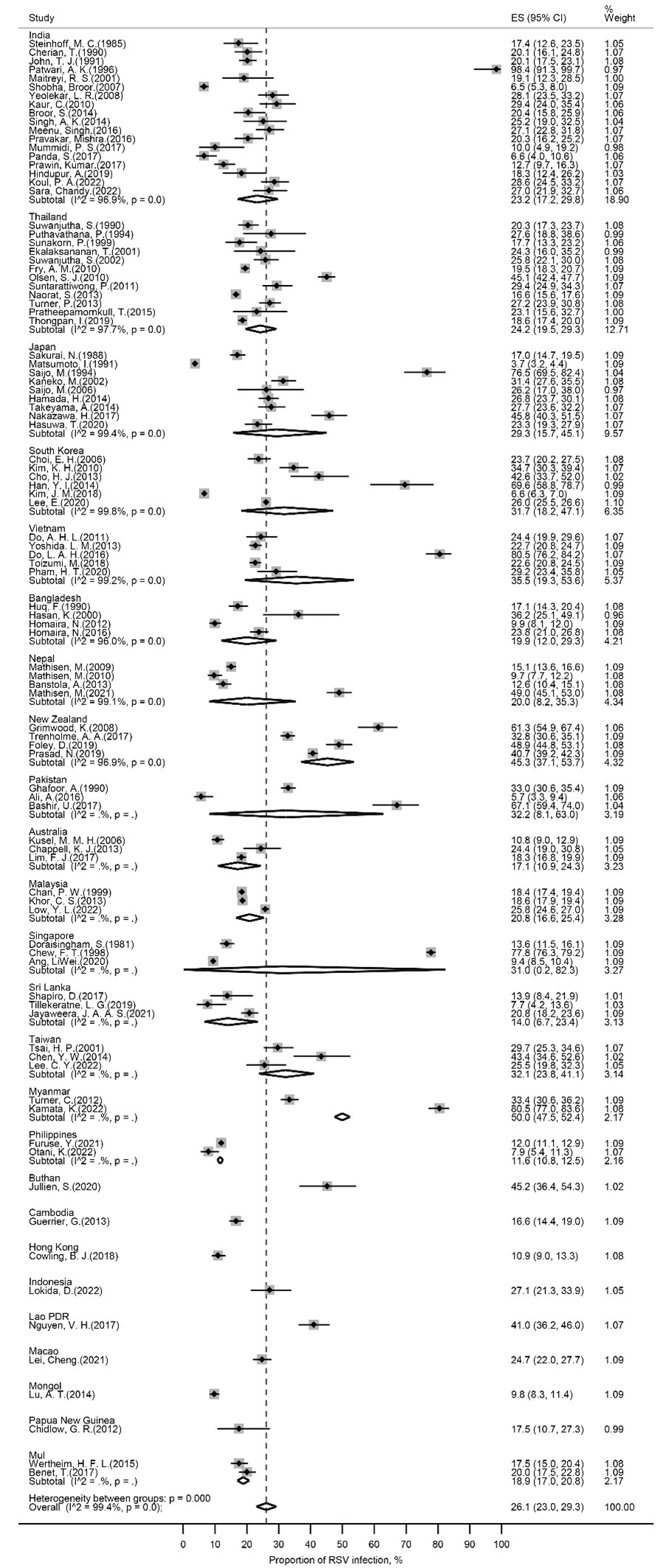

Supplement: Supplementary file 3 — Figure S2 Proportion of RSV in Western Pacific and Southeast Asia Regions excluding China [file IRV-19-e70077-s002.jpg]
